# Diverted from Landfill: Reuse of Single-Use Plastic Packaging Waste

**DOI:** 10.3390/polym14245485

**Published:** 2022-12-15

**Authors:** Kit O’Rourke, Christian Wurzer, James Murray, Adrian Doyle, Keith Doyle, Chris Griffin, Bernd Christensen, Conchúr M. Ó Brádaigh, Dipa Ray

**Affiliations:** 1 School of Engineering, Institute for Materials and Processes, The University of Edinburgh, Sanderson Building, Robert Stevenson Road, Edinburgh EH9 3FB, UK; 2 School of GeoSciences, UK Biochar Research Centre, The University of Edinburgh, Edinburgh EH9 3FF, UK; 3 Materials Research Institute, Technological University of the Shannon, University Road, Co. Westmeath, N37 HD68 Athlone, Ireland; 4 PALTECH, Ballycumber Road, Co. Offaly, R35 XR57 Clara, Ireland; 5 Johns Manville, 10, 100, West Ute Ave., Littleton, CO 80127, USA; 6 Johns Manville Europe GmbH, Werner-Schuller-Str. 1, 97877 Wertheim, Germany

**Keywords:** mixed plastic packaging waste, recycled plastics, compression moulding, mechanical testing, fracture surface, mixed polyethylenes

## Abstract

Low-density polyethylene (LDPE) based packaging films mostly end up in landfill after single-use as they are not commonly recycled due to their flexible nature, low strength and low cost. Additionally, the necessity to separate and sort different plastic waste streams is the most costly step in plastics recycling, and is a major barrier to increasing recycling rates. This cost can be reduced through using waste mixed plastics (wMP) as a raw material. This research investigates the properties of PE-based wMP coming from film packaging wastes that constitutes different grades of PE with traces of polypropylene (PP). Their properties are compared with segregated individual recycled polyolefins and virgin LDPE. The plastic plaques are produced directly from the wMP shreds as well as after extruding the wMP shreds into a more uniform material. The effect of different material forms and processing conditions on the mechanical properties are investigated. The results of the investigation show that measured properties of the wMP fall well within the range of properties of various grades of virgin polyethylene, indicating the maximum possible variations between different batches. Addition of an intermediate processing step of extrusion before compression moulding is found to have no effect on the tensile properties but results in a noticeably different failure behaviour. The wMP does not show any thermal degradation during processing that was confirmed by thermogravimetric analysis. The results give a scientific insight into the adoption of wMP in real world products that can divert them from landfill creating a more circular economy.

## 1. Introduction

Thermoplastic polymers are particularly appealing to use due to their recyclability over thermosetting polymers. Recently, the demand for recycled materials has increased due to government legislation and many companies switching to recycled materials to improve their brand sustainability image. In 2017, over 90% of plastics produced globally were derived from virgin material made using fossil fuels [1], however, the current climate crisis has forced a necessary movement away from the use of fossil fuels. In order to keep up with the current demand of plastics, it is necessary to find a more efficient way to utilise the plastics already in circulation. Furthermore, the current recycling rate must be increased to meet recycling targets, such as the EU target for recycling 50% of plastic packaging waste by 2025 and 55% by 2030 [2]. The necessity to separate and sort different plastic waste streams is the most labour-intensive and costly step in any plastics recycling process and one of the main barriers to increasing recycling rates.

Currently, plastic packaging consisting of high-density polyethylene (HDPE), polypropylene (PP), and polyethylene terephthalate (PET) are commonly recycled. However, a large proportion of single-use plastics packaging wastes that consists primarily of low-density polyethylene (LDPE), are not generally recycled due to their flexibility, low mechanical performance and low cost [3]. Within the UK, it is estimated that five million tonnes of plastics are used every year [4], with 67% of this consisting of plastic packaging [5]. However, LDPE films and bags are not collected with household recycling in the UK, an addition which could significantly increase recycling rates.

Waste mixed plastics (wMP) originating from single-use plastics packaging consists mostly of various grades of polyethylene including LDPE and HDPE. Such wMP have the potential to offer mechanical properties superior to LDPE alone. In addition, using such wMP can eliminate the need for separating the plastics in the recycling stream saving cost.

There is an abundance of published literature focusing on the use of recycled plastics, investigating polyolefin blends such as PE and PP (both virgin and recycled) [6,7,8,9,10,11,12,13,14,15,16], and studying the properties of various blends of virgin and recycled PE [17,18,19,20,21,22,23,24,25,26,27,28]. However, there is a noticeable gap in the literature characterising waste mixed plastics comprising of different grades of PEs and PPs of unknown composition.

Al-Attar [17] investigated virgin LDPE/LLDPE (vLDPE/vLLDPE) blends. The study showed that incorporating vLLDPE into vLDPE results in higher mechanical properties than vLDPE alone. This correlated with the research carried out by Cho et al. [29] who reported synergistic characteristics in vLLDPE/vLDPE blends, which exhibited higher tensile strength at yield and elongation at break than the vLLDPE, despite the lack of co-crystallisation in the blends. Similarly, Luyt et al. [20] investigated the thermal and mechanical properties of vLLDPE/vLDPE/wax ternary blends, noting that as the proportion of vLDPE in the blend increases, the mechanical properties decrease due to the decreasing crystallinity.

Rana [18] investigated the variation in properties by increasing vLLDPE content in vLLDPE/vHDPE blends. A minimal variation was revealed in mechanical properties between the blends containing between 20%–80% vLLDPE, with the plateaued values slightly lower than that of 100% vHDPE. This study is promising considering the natural material fluctuation in collected recyclable plastics; however, both vLLDPE and vHDPE have much more linearly packed polymer chains than vLDPE, which makes up a large proportion of waste plastic packaging films.

When utilising wMP, a considerable variation in material type is expected, and also variations in grades of materials, their molecular weights, and proportion of each material in each batch of mixed waste. Bai et al. [19] investigated the effect of molecular weight (MW) on the mechanical and rheological properties of HDPE blends. The high MW samples had superior impact strength, but the impact strengths of the blends were lower than the rule of mixtures theoretical values. However, the authors observed that the miscibility in the melt state and the solid-state differs and that during cooling and crystallisation there is likely to be phase separating, resulting in poor mechanical properties.

The studies investigating polyethylene blends mentioned previously have only considered the effects on virgin materials. There has been some studies on blending recycled PEs/polyolefins in different proportions. Yousif et al. [24] studied different blends of recycled LDPE (rLDPE), rHDPE, and rPP. Blending the three with different proportions was shown to improve the tensile stress up to 14–25% higher than the individual polymers alone, confirming the prospective increase in recycled plastics quality through blending.

Although many virgin PEs are shown to have better mechanical properties than their recycled counterpart, if higher properties are required, there is the potential of making blends containing both virgin and recycled PEs. For example, Cecon et al. [22] investigated the effect of adding different amounts of post-consumer recycled polyethylene (PCRPE) on virgin polyethylenes of different densities (vLDPE, vLLDPE, vMDPE, and vHDPE). Both LDPE and LLDPE blends displayed increases up to 75% in the tensile modulus and 56% in the yield strength compared to those without PCRPE. MDPE and HDPE blends, however, presented decreases up to 70% and 56% in tensile modulus and yield strength, respectively, compared to their virgin counterparts, which is expected given the lower crystallinity of the blends.

With the exception of Cecon [22] and Yousif [23], there have not been many other studies focusing on the characterisation of recycled LDPE blends. Additionally, Cecon [22] blended recycled and virgin polymers together, which likely gives an overestimation of the expected properties of rPE blends. Mihrabi-Mazidi et al. [30] characterised rHDPE and rPP blends both of which have higher mechanical properties than LDPE, and Yousif’s investigation [23] focused only on the tensile properties. These studies are not characteristic of a true recycling stream which, without segregation, could consist of LDPE, HDPE, LLDPE, PP, PET, and many more thermoplastics. Furthermore, these studies investigated uniformly mixed blends, through extrusion or injection moulding, rather than utilising the polymers as-received, which adds cost to the recycling process. The lack of comprehensive studies focusing on waste LDPE blends must be addressed if plastic packaging wastes are to become more widely recycled, given the high proportion of LDPE in plastics packaging.

The lack of comparative studies considering recycled polyolefin blends and virgin polyolefin properties, highlights the need for a benchmark recycled material database. This would be crucial in order to compare and understand the difference in using waste mixed plastics over segregated recycled plastics.

This present study characterised waste mixed plastics from packaging film wastes alongside segregated recycled and virgin plastics. The different types of plastics present in the wMP was determined using differential scanning calorimetry. The thermal stability of the materials was determined using thermogravimetric analysis to ensure the materials do not undergo thermal degradation during the processing steps. The processing was carried out using wMP shreds in the as-received condition, as well as after an intermediate extrusion step. The processing parameters were varied to understand their effects on the mechanical properties. The tensile, flexural, Izod impact tests were carried out to compare the material properties. Scanning electron microscopic (SEM) images were taken to assess the tensile fracture surfaces of the materials.

## 2. Experimental

### 2.1. Materials

**Figure 1:**
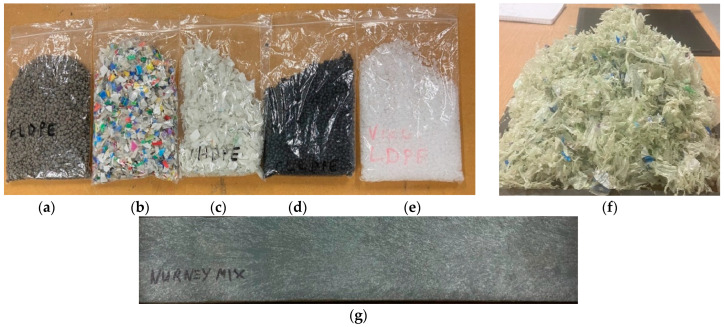
(**a**) rLDPE pellets, (**b**) rPP flakes, (**c**) rHDPE flakes, (**d**) rLLDPE pellets, (**e**) vLDPE pellets and (**f**) shredded wMP and (**g**) extruded block.

The primary material of focus in this study is waste mixed plastics (wMP), washed, shredded, and supplied by PALTECH (Polymer Alloy Technology, Clara, Ireland) [31]. PALTECH takes the plastic wastes originated from food packaging from Tesco Ireland stores (Dublin, Ireland) [32]. These mixed plastics packaging wastes undergo one stage of sorting, which is float-sink separation. The wMP that float on water are collected by PALTECH, therefore these contain mainly polyolefins, having densities lower than water. In addition to the wMP, segregated individual recycled polyolefins were also investigated such as recycled low-density polyethylene (rLDPE) and recycled linear low-density polyethylene (rLLDPE), both in pellet form; recycled high-density polyethylene (rHDPE) and recycled polypropylene (rPP), both in the form of flakes; and virgin low-density polyethylene (vLDPE) in pellet form (supplied by PLASTISERVE Ltd., Leeds, UK). The segregated individually recycled polyolefins will be referred to as “recycled plastics” throughout the remainder of the paper. As this study investigates recycled plastics, the materials used are likely to be a blend of multiple grades of each segregated plastic. The materials used in this study are shown in Figure 1. A block of extrusion moulded wMP, measuring 400 × 10 × 1.5 mm, was also supplied by PALTECH to compare with the wMP shreds (Figure 1g) and this is referred as ‘extruded block’ in the rest of the manuscript.

### 2.2. Manufacturing

As shown above in Figure 1, the starting materials were in different forms; shredded and pellets. These materials were directly made into plaques by compression moulding. An additional plaque was produced by using the extruded block of the shredded wMP (Figure 1g). This will give a direct comparison if an intermediate extrusion step is required to melt-mix the shredded wMP into a uniform material before converting them into products by compression moulding. The elimination of any intermediate processing step could save money and facilitate the reuse and recycling of such low value plastics wastes that generally end up in landfill.

**Figure 2:**
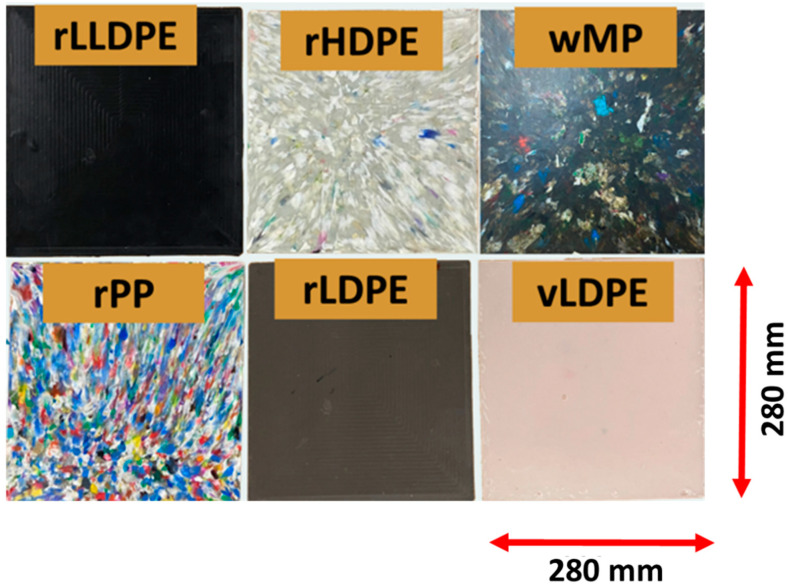
Manufactured plaques (280 mm × 280 mm × 3 mm) made from various recycled plastics, vLDPE, and wMP.

Plastic plaques were manufactured by compression moulding with a PEI lab 450 hydraulic press, using a two-part closed mould (Figure S1). The plastic plaques were of dimensions 280 mm × 280 mm × 3 mm (Figure 2). The processing cycle used in the hydraulic press was as follows:

1. Heating from 20 °C to 180 °C at a rate of 10 °C/min.
2. Holding at 180 °C for 10 min at 2 bar pressure.
3. Cooling from 180 °C to 20 °C at 10 °C/min at 2 bar pressure.

Some plaques had to undergo additional processing cycles in order to include enough material in the mould, thereby ensuring that the material quantity is consistent across all plaques. The amount of additional cycles required for each material varied due to the difference in the bulk densities of the different material forms (Table 1).

Three sets of studies were carried out with the wMP plaques. In the first set, wMP were characterised in comparison to both the recycled plastics and vLDPE. In the second set, the effect of processing pressure was studied on the quality and the mechanical properties of wMP plaques. The third set compared the mechanical properties of wMP plaques manufactured using two different processing methods; compression moulding of wMP shreds, and compression moulding of the extruded wMP block. The three sets of samples manufactured are described in Table 1.

### 2.3. Test Methods

#### 2.3.1. Density

The density of each plaque was measured using an Ohaus density determination kit. Six samples were measured from each set. The following equation was used to determine the density of each sample, and an average was taken for each plaque.

$$d_{sample}=\frac{mass\;in\;air\;\left(g\right)}{mass\;in\;distilled\;water\;\left(g\right)}\times d_{distilled\;water}\left(\frac{g}{{\mathrm{cm}}^{3}}\right)$$

#### 2.3.2. Differential Scanning Calorimetry

Differential scanning calorimetry (DSC) analysis was performed on 5 random wMP shred samples to identify the various plastics present in it, and to determine the processing temperature. The analysis was carried out using a Perkin Elmer Pyris Thermal analyser, in a nitrogen atmosphere from 25 °C to 200 °C at a heating rate of 10 °C/min.

#### 2.3.3. Thermogravimetric Analysis

Thermogravimetric analysis (TGA) was carried out with a Mettler Toledo, USA TGA/DSC 1 analyser between 25 °C to 600 °C at a rate of 10 °C/min in a nitrogen atmosphere to determine the thermal stability of the samples over that temperature range. TGA results were also used to understand if there is any effect from any additives present in the wMP/recycled plastics on their thermal degradation behaviour. To observe the thermal stability of the material at the processing temperature (180 °C), an isothermal TGA cycle was run from 25 °C to 200 °C in air at a rate of 10 °C/min and held at this temperature for 1 h before cooling back to 25 °C.

#### 2.3.4. Tensile Testing

Tensile testing was used to determine the tensile strength and modulus of each type of plastic. The tests were carried out following ASTM D638, using six dumbbell-shaped specimens of Type IV from each plaque. Each sample was loaded at a speed of 5 mm/min until failure, and the values for the load and extension were recorded using the Bluehill^®^ testing software (version 3.61).

#### 2.3.5. Flexural Testing

Flexural testing was carried out following ASTM D790 (American Society for Testing and Materials, West Conshohocken, PA, USA), using six samples from each plaque with dimensions of 61 mm × 13 mm × 3 mm. The testing speed used was 1.3 mm/min and the span-to-thickness ratio was 16:1 following the standard.

#### 2.3.6. Impact Testing

Izod impact testing was carried out to determine the impact strength of the plastic samples. This was carried out following ASTM D256 (American Society for Testing and Materials, West Conshohocken, PA, USA) and using a CEAST 6545 impact tester with a 5.5 J pendulum.

#### 2.3.7. Microscopy

Scanning electron microscopy (SEM) (JEOL, Tokyo, Japan) was used to examine the tensile fracture surface of the specimens using a JEOL JSM series instrument. Each sample was prepared by gold spluttering to increase the conductivity, and the voltage used to observe the samples was 15 kV. Scanning electron microscopy was also used to see the quality of consolidation of each plastic plaque.

#### 2.3.8. Statistical Analysis

The results were analysed using statistical methods to determine the significance of the tensile and flexural test results. As this study focuses on mixed waste plastics, the types of plastics present per batch varies. A statistical *t*-test can be used to determine whether there is a significant difference in the means of two groups of values [33]. Therefore, the *t*-test method (unequal variance) was used to determine whether the difference in the test results was due to the investigation or due to the random nature of using mixed plastics. The equation used to calculate the *t*-test value is as follows:

$$t=\frac{\left(x_{1}-x_{2}\right)}{\sqrt{\frac{{\left(s_{1}\right)}^{2}}{n_{1}}+\frac{{\left(s_{2}\right)}^{2}}{n_{2}}}\;}$$

where:

*t* = *t*-test value

*x* = sample set mean

s = standard deviation

*n* = number of samples

Higher *t*-test values indicate that there is a significant difference between the two groups of values. A small *t*-test value indicates that the groups are similar, and there is no statistically significant difference between the means. From the *t*-test, a *p*-value is determined through comparison tables. If the corresponding *p*-value is less than a chosen value, for example α =0.05 for a 95% confidence interval, or α = 0.01 for a 99% confidence interval, then the means of the two groups are statistically different.

## 3. Results and Discussion

The wMP samples were first characterised with DSC and TGA to identify the types of polymers present and to understand their thermal stability.

### 3.1. Thermal Characterisation

#### 3.1.1. Characterisation by DSC

**Figure 3:**
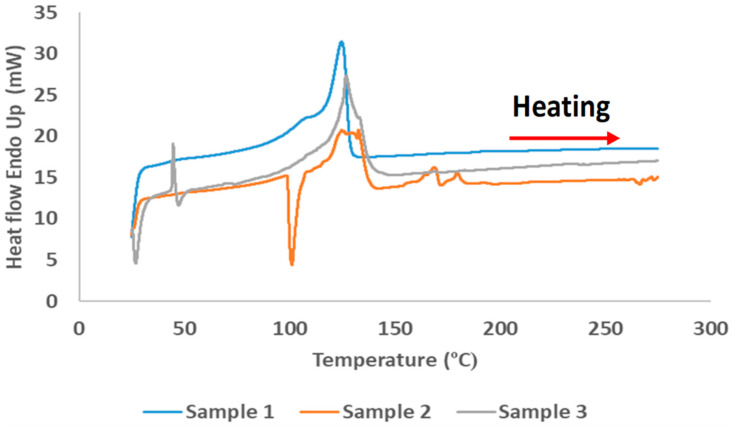
DSC thermograms showing the heat flow for three samples of wMP.

Five wMP samples were randomly selected and subjected to DSC. Three representative DSC curves of the wMP samples are shown in Figure 3. The melting was observed mostly between 105–135 °C, with small melting traces at 170 °C in one of the samples. These results allowed the processing temperature to be set at 180 °C.

The melting peaks, and the typical melting ranges of different grades of polyethylenes and polypropylene observed in literature are shown in Table S1. The plastics identified in the DSC thermograms of wMP are primarily different grades of PE, with a small trace of PP in one of the samples.

The DSC data was used to calculate the crystallinity of the as-received plastic samples. The average crystallinity values are shown in Table S2. The average crystallinity of the wMP samples was calculated as around 40%. The wMP comprises of different grades of polyethylene that are commonly used as packaging films. Though some HDPE might be present, the wMP is mostly composed of different grades of LDPEs. Hence, the average crystallinity of wMP was found to be very similar to the LDPE samples, both recycled and virgin.

#### 3.1.2. Thermogravimetric Analysis

**Figure 4:**
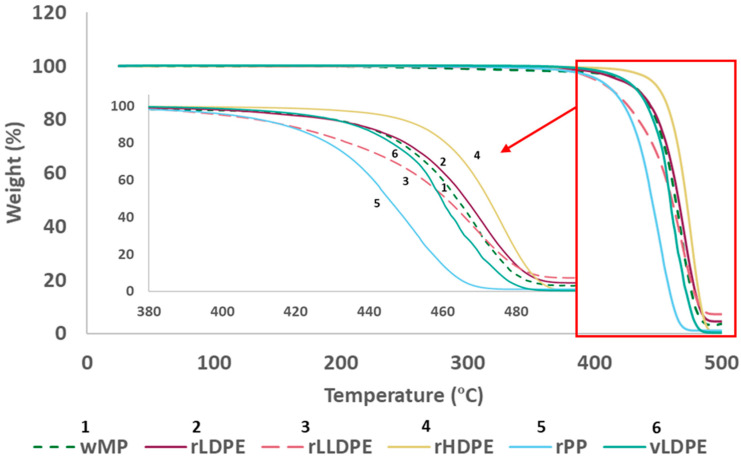
Thermal degradation profile of all materials from 25–600 °C in nitrogen.

The TGA curves in Figure 4 show that the wMP has a marginally lower degradation onset temperature than the other recycled plastics tested, at around 350 °C. This is however much higher than the processing temperature of 180 °C, and hence not likely to cause any thermal degradation during processing.

The isothermal TGA results observed after holding the samples at 200 °C for 1 h in air showed no loss in weight (Figure S2). This indicates that the processing temperature at 180 °C is not likely to cause thermal degradation of any of the materials. This also indicates that there should be no effect on the mechanical properties of the plaques that experienced additional processing up to 4 cycles.

### 3.2. Tensile Testing

The tensile properties of the samples, described in Table 1, are given below.

#### 3.2.1. Comparison of wMP, Recycled Plastics, and vLDPE

**Figure 5:**
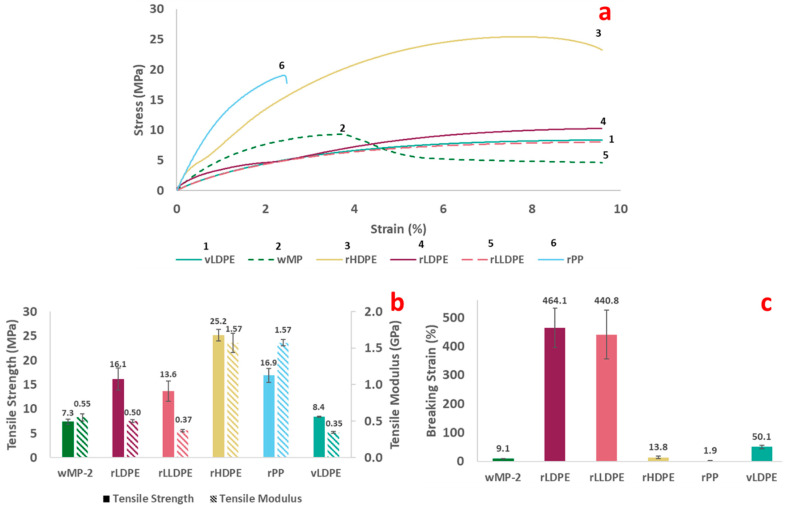
Graphs showing (**a**) representative tensile stress–strain curves, (**b**) tensile strength and tensile modulus, and (**c**) breaking strain of wMP-2, recycled plastics and vLDPE.

The representative stress–strain curves, the tensile properties, and the breaking strain of the wMP samples in comparison to recycled plastic samples and vLDPE (Table 1) are shown in Figure 5.

The tensile stress–strain curves are typical of ductile thermoplastics. The stress–strain curve of wMP-2 resembles LDPEs, but the presence of some HDPE and rPP is evident as the initial slope is higher at the start (Figure 5a).

The tensile strength and modulus of the wMP-2 and other plastics are shown in Figure 5b. The tensile strength of the wMP is found to be the lowest of all the tested materials, having a value of 7.3 ± 0.7 MPa. As mentioned before, the wMP-2 plaque was compression moulded directly from the wMP shreds and the samples were made of several shreds bonded together. As the shreds are different grades of PE, there is therefore non-uniformity across the sample, and its performance is dictated by the bonding between the different shreds. The bonding between the shreds influences the tensile strength of the material, which will be discussed later in this section. The rHDPE and rPP samples were also initially in flake form, but these were recycled materials with consistent shape and size even if the grades were different. The wMP, on the other hand, consisted of different materials, different grades of materials, different size, shape, and thickness, which created additional non-uniformity.

The tensile modulus of wMP-2 is lower than that of the rPP and rHDPE, but is higher than the LDPEs tested (Figure 5b). The increase in modulus of wMP-2 was statistically significant according to a *t*-test analysis with *p* = 0.02 and α = 0.05. This higher modulus observed in wMP-2 compared to the rLDPE, rLLDPE, and vLDPE could be to be due to the effect of rHDPE and rPP present in the mix.

The rLDPE and rLLDPE samples exhibited much higher breaking strains than the other recycled plastics (Figure 5c). The breaking strain of vLDPE is found to be lower than that of rLDPE, which might be attributed to the fact that rLDPE contains different grades of LDPE, some of which are likely to have higher mechanical properties than the single grade of vLDPE investigated in this study. This highlights the difficulty in directly comparing virgin with recycled materials, as the recycled materials are likely to contain a mix of different grades.

LDPE and LLDPE are less crystalline than HDPE and PP and can therefore extend to higher elongations. This is because the proportion of amorphous regions are higher, which means that the polymer chains in these regions are able to detangle and uncoil much more than the crystalline regions, leading to higher elongations before breaking.

The wMP-2 had the lowest breaking strain of all the materials tested except rPP. The wMP-2 shreds were pressed together during manufacture and there was no melt mixing, so the individual plastic shreds were just adhered to one another during consolidation. During tensile testing, the force required to separate the individual plastic shreds was lower than that required to stretch the individual shreds, therefore the wMP-2 samples failed with very low elongation.

**Figure 6:**
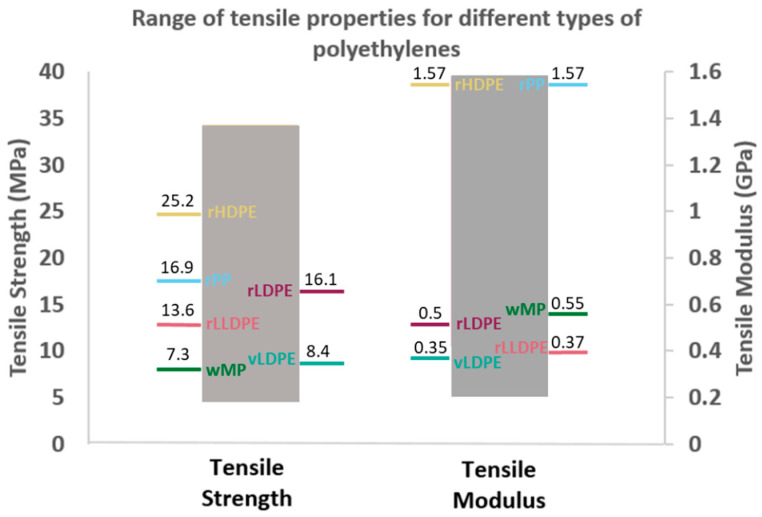
The possible range of variation of tensile strength and tensile modulus values of different types of recycled polyethylenes, polyethylene-based wMP as well as their measured values.

As wMP batches can vary, it is important to have an understanding about the range of variation possible in their tensile strength and tensile modulus values. Figure 6 shows the range of tensile strength and tensile modulus values that covers different grades of virgin PEs including HDPE. The experimentally measured tensile strength and tensile modulus values of wMP-2 and the recycled plastics fit well within that range, as shown in Figure 6. Any variation in the composition of wMP, originating from the PE-based packaging film wastes, are likely to vary between these two ranges.

#### 3.2.2. Comparison of wMP Compression Moulded under Different Pressures

**Figure 7:**
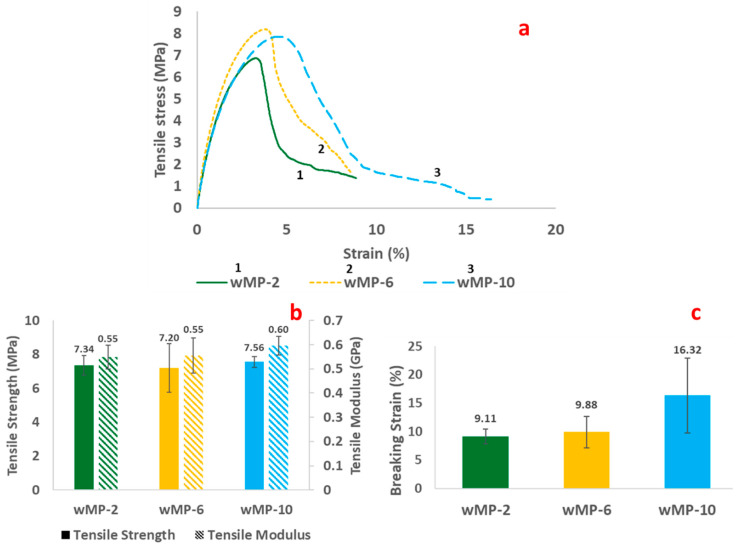
(**a**) representative tensile stress–strain curves, (**b**) tensile strength and tensile modulus, and (**c**) breaking strain of wMP processed by compression moulding at different pressures.

The tensile results of the wMP samples manufactured under three different consolidation pressures are shown in Figure 7. The tensile results indicate that increasing the consolidation pressure of the shredded wMP from 2 bar to 10 bar did not influence the tensile strength of the wMP samples. Increasing the pressure was found to increase the tensile modulus by 8% (from 2 bar to 10 bar), but this is not a statistically significant result, with *p* = 0.26 and α = 0.05.

The average breaking strains of the three sets of samples show that increasing the pressure from 2 to 10 bar increases the breaking strain by 79%. This result was not statistically significant for *p* = 0.06 and α = 0.05, and the increase might be due to the random proportion of different materials in the mix.

**Figure 8:**
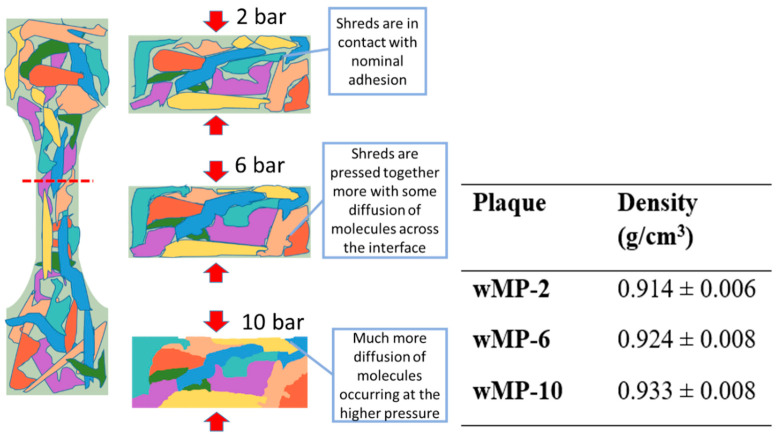
Different extents of adhesion between individual wMP shreds due to different consolidation pressures leading to changes in the densities.

A schematic is shown in Figure 8 displaying the possible effects that the consolidation pressure might have on the compaction of the shredded plastics during compression moulding. This schematic is based on the measured density values of the samples, as shown in Figure 8. The higher densities indicate a higher level of compaction at higher consolidation pressures, but that did not influence the tensile properties of the wMP.

During compression moulding, the wMP shreds melt but they do not flow as happens in the case of extrusion or injection moulding. Under heat and pressure, the molten material at the interface of two adjacent shreds fuse together. At higher pressures, it is easier for the shreds to pack together, facilitating diffusion of molecules from one molten shred to the adjacent one, allowing for higher compaction and increased density.

#### 3.2.3. Comparison of wMP Manufactured Using Different Processing Routes

**Figure 9:**
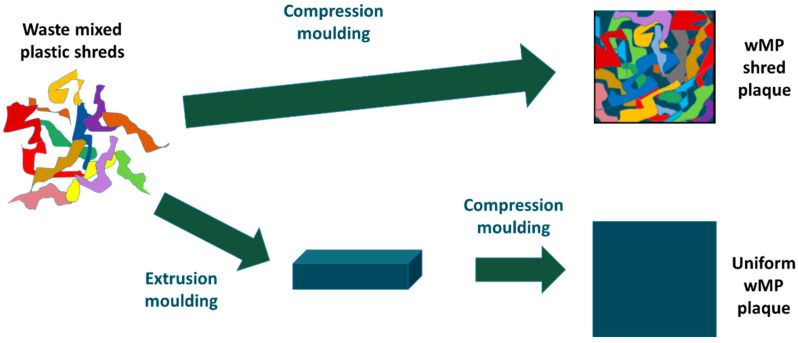
Schematic showing the different processing routes of manufacturing the wMP plaques.

The wMP plaques reported above were manufactured by compression moulding of wMP shreds. As mentioned in Section 2.1, an equivalent wMP plaque was prepared by compression moulding a pre-extruded wMP block. This block was prepared by extrusion moulding the wMP shreds into the form of a block. A piece was cut from this extruded block and compression moulded into a plaque of equal weight and dimensions as the previous wMP plaque, and using the same processing parameters. This is shown schematically in Figure 9.

**Figure 10:**
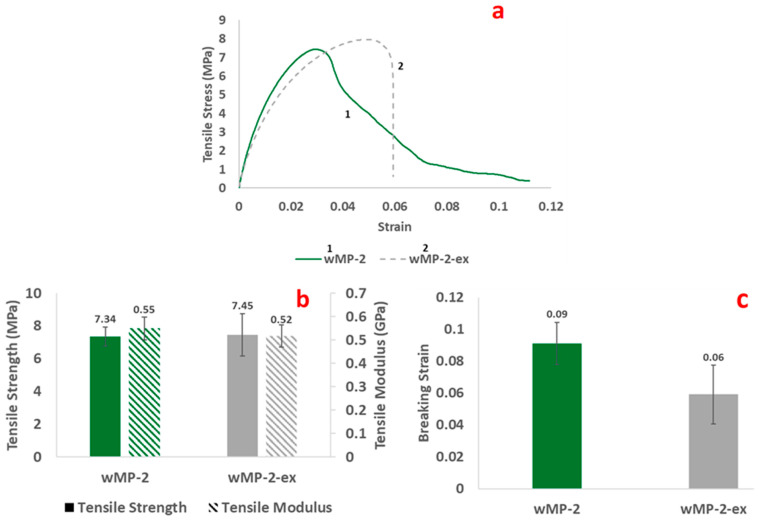
(**a**) Representative tensile stress–strain curves, (**b**) tensile strength and tensile modulus, and (**c**) breaking strain of wMP manufactured with different processing methods.

The tensile results of the two sets of samples are shown in Figure 10. The effect of different processing methods on the tensile properties of wMP are discussed below.

It is evident that the failure behaviours are different in the two samples (Figure 10a). The wMP-2 exhibited a gradual drop in load after reaching the peak, resulting in a higher breaking strain (0.0911). While the drop in load in wMP-2-ex was sudden, resulting in a lower breaking strain value (0.0591). The higher peak load and sudden drop in load in wMP-2-ex samples indicates that the sample behaved more like a uniform single material and was able to sustain a higher load before failure.

The tensile strength values for both the samples are quite similar, as shown in Figure 10b. The wMP-2 has a marginally higher tensile modulus, but this value is not statistically significant, with *p* = 0.86 and α = 0.05. These results indicate that an intermediate extrusion step before compression moulding might not have any additional benefit on the tensile strength and tensile modulus values of the shredded wMP materials. Any intermediate processing step adds a cost to the process, which is not desired while reprocessing such low-cost waste materials. These results therefore indicate that shredded wMP can be directly used for producing compression moulded products. The difference in failure behaviours is explained schematically in Figure S3.

The higher strength wMP shreds in the gauge length of the tensile test specimens get stretched during tensile testing when the weaker shreds break, or the shreds are pulled out from each other. This stretching, breaking and pulling-out of individual plastic shreds one after another causes the gradual failure of the sample (Figure S3a).

On the other hand, the wMP-2-ex samples were uniformly mixed and the failure happened suddenly when the sample fractured as a single material, as shown in Figure S3b.

### 3.3. Flexural Testing

**Figure 11:**
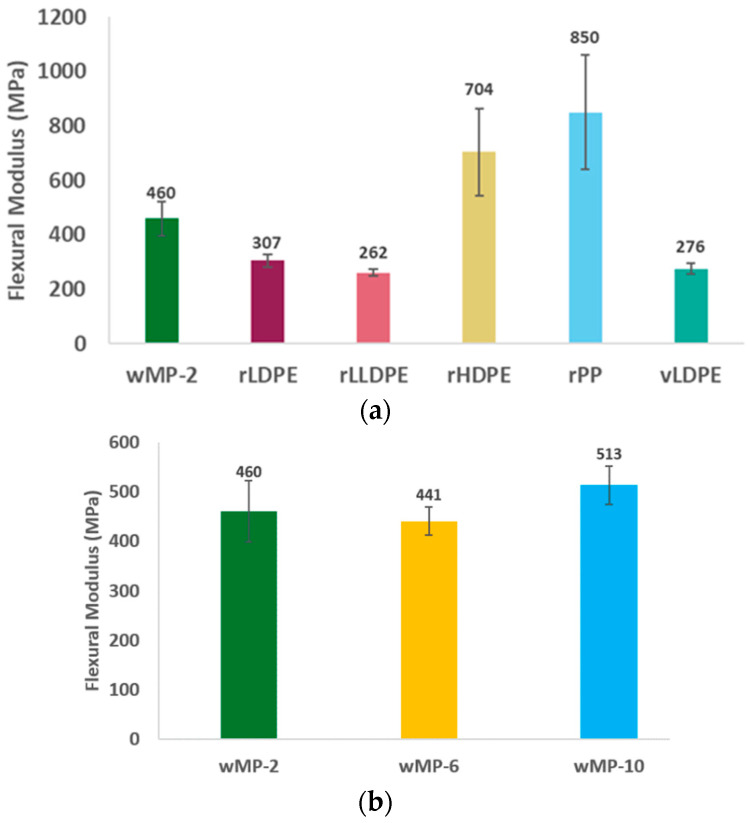

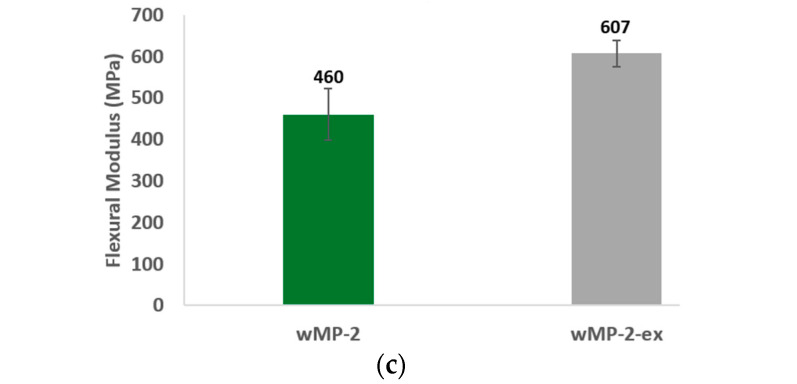
Flexural modulus of wMP (**a**) compared to recycled plastics and virgin LDPE, (**b**) compression moulded under different pressures, and (**c**) manufactured using different processing routes.

The flexural test results of the investigated materials described in Table 1 are given in Figure 11 below.

#### 3.3.1. Comparison of wMP, Recycled Plastics, and vLDPE

Similarly to the tensile modulus, the flexural modulus of the wMP-2 samples is lower than rHDPE and rPP, but is noticeably higher than the other LDPEs, about 33% and 40% higher than rLDPE and vLDPE, respectively (Figure 11a). The flexural modulus of the wMP is determined by the inherent rigidity of the individual shreds. As wMP contains some proportions of HDPE and PP, which have higher crystallinity, it is expected that the wMP flexural modulus will be higher than the other LDPEs. That is evident in Figure 11a. In flexural testing, both compressive and tensile forces act on the specimens. Due to the nature of the loading, the possibility of the individual shreds being debonded from each other during testing is less pronounced here than that in the tensile testing.

#### 3.3.2. Comparison of wMP Compression Moulded under Different Pressures

Figure 11b indicates that increasing the consolidation pressure did not affect the flexural modulus of the wMP samples. During three-point bending, the applied force is compressive on the top side while tensile on the bottom side of the sample. As mentioned above, the applied tensile force might not be large enough to separate the shreds as seen in the case of a tensile test. Therefore, the flexural modulus was not much influenced by the level of compaction and hence, the level of adhesion between the adjacent wMP shreds was not the dominant factor. This is supported by the test results as the flexural modulus values of the samples manufactured under different consolidation pressures were very similar.

#### 3.3.3. Comparison of wMP Manufactured Using Different Processing Routes

The flexural modulus of wMP-2-ex is 32% higher than that of wMP-2, which is statistically significant for a *p*-value of 0.0006 and α = 0.001. Therefore, it is clear that an intermediate extrusion step before compression moulding can significantly increase the flexural modulus. This can be attributed to the fact that the wMP-2-ex sample is much more uniform than the wMP-2. During three-point bending, the sample experiences both tension and compression, and in tensile testing there are only tensile forces. Therefore, it might be reasonable to assume that extruding the wMP is advantageous for increasing compressive properties.

### 3.4. Impact Testing

The impact test results of the investigated materials are given in this section.

#### 3.4.1. Comparison of wMP, Recycled Plastics, and vLDPE

The determination of the impact strength relies on the sample failing under impact. Many of the materials tested for impact (rLDPE, vLDPE, rLLDPE) did not break with the applied 5.5 J pendulum and a comparison has been made based on the observed results.

The wMP-2 samples exhibited an impact strength 41% higher than rPP and 74% lower than rHDPE (Figure S4). It is clear from the error bars that there is a wide variation in the values measured for rHDPE, but the impact strength of rHDPE was confirmed to be statistically different from wMP-2 and rPP. The impact testing results showed that rLDPE had a higher impact strength than rHDPE as the rLDPE sample did not break during testing. As the highest proportion of polymer in wMP is LDPE, it was therefore expected that the wMP-2 sample would also have a higher impact strength than rHDPE. The average impact strength of wMP-2 was in fact lower, therefore the inclusion of other plastics in wMP-2 increased the brittleness of the sample compared to LDPE alone, reducing the impact strength. This agrees with results found in the literature, such as Shebani’s investigation [28] which showed that the impact strength of vHDPE/vLDPE blends increased as the LDPE content increased.

#### 3.4.2. Comparison of wMP Compression Moulded under Different Pressures

The impact strength comparison of wMP manufactured under different consolidation pressures is shown in Figure S5.

The impact strength of wMP samples at each pressure appears to be similar, with the average impact strength of wMP-2 only marginally lower. It was confirmed through statistical *t*-test that increasing the pressure has no effect on the impact strength, with *p* = 0.26 and α = 0.05.

#### 3.4.3. Comparison of wMP Manufactured Using Different Processing Routes

The impact strength of wMP-2 and wMP-2-ex were compared. The wMP-2-ex sample did not break during impact testing and so the impact strength could not be measured, as shown in Figure S6.

LDPE is a ductile and low modulus plastic with high toughness, so is unlikely to break during impact testing. As wMP are mainly composed of LDPE, it was expected that these samples also would not break, however this was not the case. It is possible that the wMP-2 sample only broke under impact due to the voids present at the interface of the individual plastic shreds, which created weak areas in the material. As the voids in wMP-2-ex are much smaller and more distributed, the voids did not impact the material breaking during testing. The voids are shown in SEM images in Section 3.5.2.

### 3.5. Fracture Surface Analysis

#### 3.5.1. Comparison of wMP, Recycled Plastics, and vLDPE

**Figure 12:**
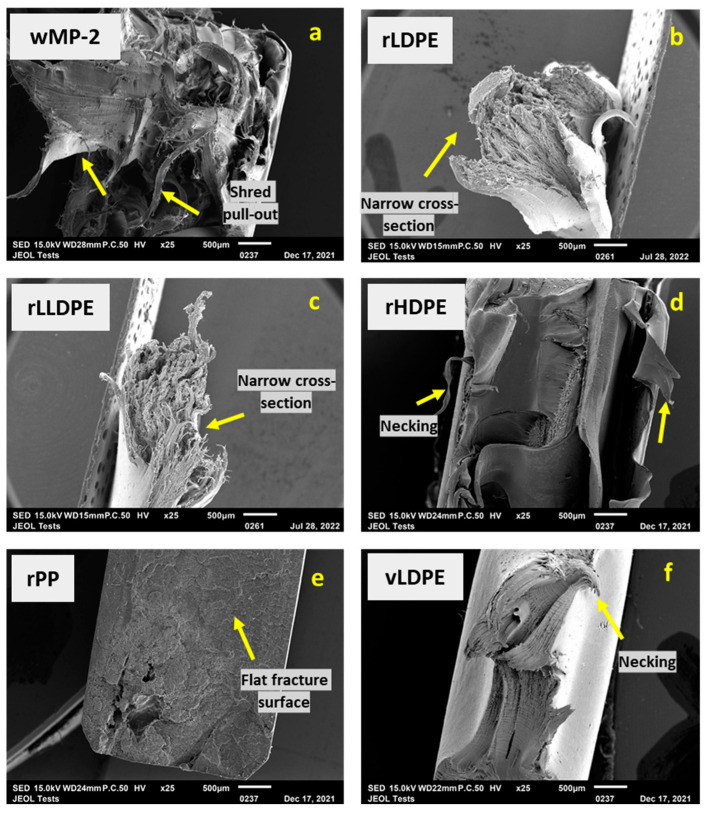
SEM images of the fracture surface of tested tensile specimens at ×25 magnification for (**a**) wMP-2, (**b**) rLDPE, (**c**) rLLDPE, (**d**) rHDPE, (**e**) rPP, and (**f**) vLDPE; showing varying degrees of stretching and pull-out of individual shreds in wMP, and narrow cross section of rLDPE and rLLDPE.

Figure S7 shows the macroscopic tensile failure behaviour of the representative samples and Figure 12 shows the SEM micrographs of the tensile fracture surfaces of the samples.

In wMP-2 (Figure S7a and Figure 12a), it is observed that some plastic shreds elongated more than others, which is likely due to the presence of different grades of PE shreds. Some shreds experience higher pull-outs and stretching during testing than others. Both rLDPE and rLLDPE showed high elongation during tensile testing as shown in Figure S7b,c. It can also be seen that both rLDPE and rLLDPE have much narrower cross-sections than the other samples, which is due to the large amount of necking that occurred during tensile testing (Figure 12b,c). rHDPE shows some elongation before failure, as observed in Figure S7d and Figure 12d. From Figure S7e and Figure 12e, it is clear that rPP has a nearly flat fracture surface without much elongation vLDPE exhibited some extent of necking, as shown in Figure S7f and Figure 12f, but not as high as rLDPE and rLLDPE.

The higher magnification SEM images of the tensile fracture surfaces in Figure S8a–d look quite different. Ductile deformations are clearly visible in rHDPE and varying degrees of stretching is visible in wMP. The SEM images of rLDPE and rLLDPE under higher magnification (Figure S8b,c) show that both samples appear very similar to wMP-2 rather than vLDPE (Figure S8f). Although the fracture surface of rPP appeared mostly flat in Figure S7e and Figure 12e, some micro ductility was observed in Figure S8e. The fracture surface of vLDPE exhibited a lamellar structure that is different to the other samples, which was also not observed in the rLDPE sample (Figure S8b), which appears more fibrillar. This could be due to the difference between virgin and recycled LDPEs or due to the grade of the vLDPE.

#### 3.5.2. Comparison of wMP Manufactured Using Different Processing Routes

**Figure 13:**
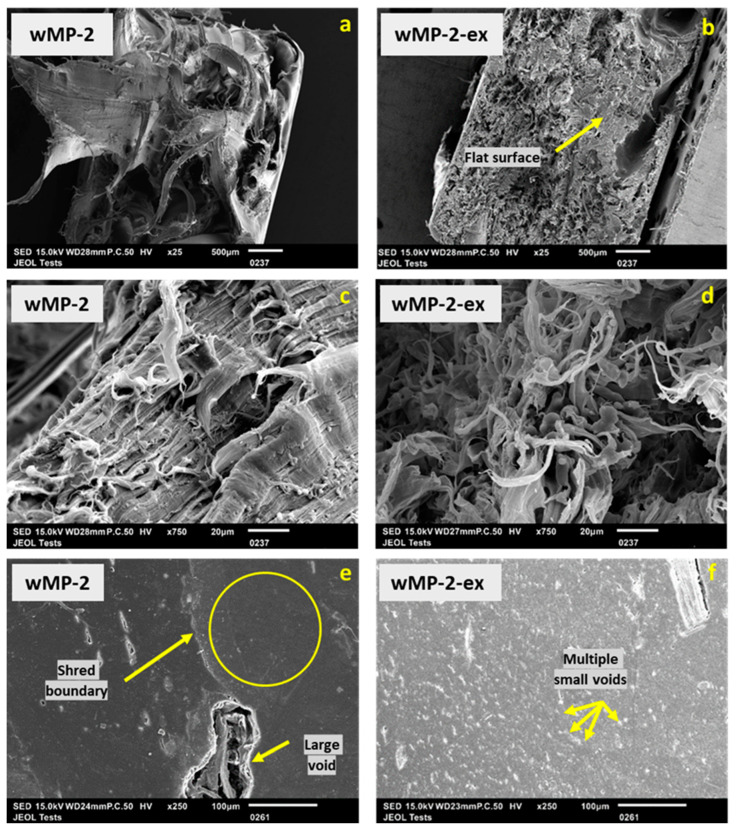
SEM images of the tensile fracture surfaces of wMP-2 and wMP-2-ex at (**a**,**b**) ×25 magnification, (**c**,**d**) ×750 magnification, and cross-sections of wMP-2 and wMP-2-ex at ×250 magnification (**e**,**f**).

The tensile fracture surfaces of the wMP-2 and wMP-2-ex samples are shown in Figure 13.

The tensile fracture surface appeared more flat for wMP-2-ex compared to that of wMP-2 (Figure 13), which is characteristic of a sudden failure (Figure 10a). At a higher magnification (×750), both samples looked quite similar (Figure 13c,d). This similarity at a microscopic level could explain the comparable tensile strength despite of the contrasting failure behaviour.

There are voids visible in both wMP-2 and wMP-2-ex samples (Figure 13e,f), but the size and distribution of the voids are very different. It is evident from Figure 13e that the voids present in wMP-2 are bigger in size and mostly concentrated at the boundary between different wMP shreds, and the areas within individual shreds are relatively free of voids. This is not the case with wMP-2-ex, as shown in Figure 13f. Here, the voids are much smaller, but numerous in number and there is no area free of voids.

This difference is expected, as the voids in wMP-2 are more likely to be found between the different shreds, especially considering the different shrinkage rates of different plastics. Within the shreds themselves there is less chance of voids as each shred is composed of just one type of plastic. Conversely in the wMP-2-ex, the wMP is mixed and no large voids are present as there are no individual shred boundaries. However, in extrusion, there is still shrinkage to consider, and air pockets present, which led to the formation of smaller voids.

Using the SEM images, and the ImageJ (Fiji) version 2.5 software, the average void content of the wMP-2 and wMP-2-ex samples were measured. The void content of wMP-2-ex and wMP-2 were measured as 0.8% and 1.8%, respectively. It was therefore observed that although the void contents were not very different in wMP-2 and wMP-2-ex, the distribution of voids was different.

## 4. Conclusions

This research investigated the properties of polyethylene-based waste mixed plastics (wMP) originating from plastics packaging wastes, comparing its properties to individually recycled polyolefin materials and virgin LDPE. The effects of different processing techniques and processing conditions on the mechanical properties of the processed plastic plaques were also investigated. The thermal degradation of the wMP starts at 350 °C in a nitrogen environment. The wMP did not show any thermal degradation when subjected to isothermal TGA at 200 °C for 1 h in air and this indicated that the processing or reprocessing of wMP plaques at 180 °C are not likely to cause any thermal degradation of the material. The properties of the wMP fell well within the range of properties of virgin polyethylene grades (3–33 MPa tensile strength, and 0.4–1.5 GPa tensile modulus). This also gives an idea about the variation in properties that might be seen between different batches of wMP samples. The tensile strength of the wMP is found to be closer to LDPE rather than HDPE and PP. Additionally the failure strains measured in wMP are much lower than rLDPE and rLLDPE. Therefore, the high ductility of LDPE is not observed in wMP.

The tensile modulus of the wMP is 9% higher than rLDPE, and the flexural modulus is 33% higher, therefore both of these properties improve when using a mix of polyolefins/PE grades. While processing shredded plastics directly by compression moulding, the consolidation pressure was not found to have any significant effect on the mechanical properties. This is an important observation for converting wMP shreds directly into product forms via compression moulding. Addition of an intermediate processing step of extrusion before compression moulding was found to have no effect on the tensile properties. However, a noticeably different failure behaviour was observed. The extra processing step resulted in an increase in flexural modulus by 32%. The shredded plastics are joined together by fusion when subjected to compression moulding only. Whereas an intermediate extrusion step before compression moulding can introduce more intimate mixing between the shredded materials leading to a more uniform material. Thus, different processing routes can bring some d±ifferences in their overall performance regardless of similar property values. The failure of the samples is dictated by what type of loading is applied on them and how they are manufactured. This scientific understanding can help to decide what might be the optimum processing route for low-cost packaging wastes avoiding any additional processing cost.

## Figures and Tables

**Table 1:** Summary of recycled plastics, wMP, and vLDPE plaques manufactured by compression moulding.

Plaque	Material	Processing Pressure (bar)	No. of Processing Cycles
wMP-2 *	Waste mixed plastic	2	4
rLDPE	Recycled low-density polyethylene	2	2
rLLDPE	Recycled linear low-density polyethylene	2	5
rHDPE	Recycled high-density polyethylene	2	2
rPP	Recycled polypropylene	2	2
vLDPE	Virgin low-density polyethylene	2	4
wMP-6	Waste mixed plastic	6	4
wMP-10	Waste mixed plastic	10	4
wMP-ex	Compression moulding (from extruded block)	2	1

* wMP-2 denotes wMP plaque manufactured under 2 bar pressure.

## Data Availability

Data is contained within the article or supplementary material.

## Supplementary Materials

The following supporting information can be downloaded at: https://www.mdpi.com/article/10.3390/polym14245485/s1, Figure S1: Closed two-part metal mould used to manufacture plastic plaques in hydraulic press.; Figure S2: Change in weight of the samples after holding at 200 oC for 1 h in air.; Figure S3: Schematic showing the failure behaviour in (a) wMP-2 and (b) wMP-2-ex during tensile testing.; Figure S4: Izod impact strength (un-notched) of wMP-2 in comparison to recycled plastics (rPP and rHDPE).; Figure S5: Comparison of Izod impact strength (un-notched) of wMP manufactured under different pressures.; Figure S6: Comparison of Izod impact strength (un-notched) of wMP samples produced by compression moulding of wMP shreds and by compression moulding of an extruded block of wMP.; Figure S7: Representative tensile test specimens of (a) wMP-2, (b) rLDPE, (c) rLLDPE, (d) rHDPE, (e) rPP and (f) vLDPE during tensile testing with additional close up images of (g) rLDPE and (h) rLLDPE, due to the high amount of stretching.; Figure S8: SEM images of the fracture surface of tested tensile specimens at x750 magnification.; Table S1: Typical melting ranges of polyolefin materials [34,35,36,37,38].; Table S2: Crystallinity of wMP and segregated recycled thermoplastics calculated from DSC analysis.; Table S3: Onset and endset temperatures for thermal degradation of wMP and recycled plastics.
