# Supplementary material for: Diverted from Landfill: Reuse of Single-Use Plastic Packaging Waste
Source: Polymers (Basel). 2022 Dec 15;14(24):5485. doi: 10.3390/polym14245485 (PMC9785204; doi:10.3390/polym14245485)
Supplement: Supplementary file 1 [file polymers-14-05485-s001.zip › polymers-2087841-supplementary.pdf]

Supplementary document

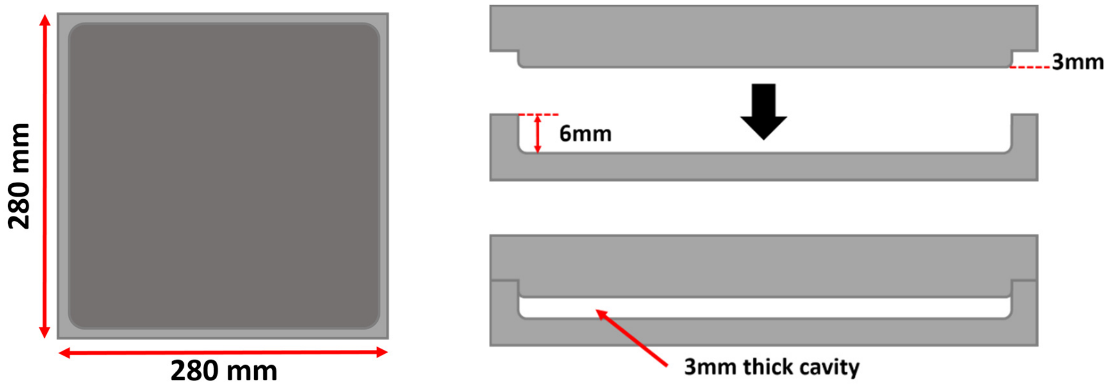

Figure S1: Closed two-part metal mould used to manufacture plastic plaques in hydraulic press.

Table S1: Typical melting ranges of polyolefin materials [1-5].

Material	Melting range (°C)
Low Density Polyethylene	105 – 123
Linear Low Density Polyethylene	120 – 127
High Density Polyethylene	122 – 137
Polypropylene	160 – 170
Waste Mixed Plastics	105 – 140 (+165 for rPP*)

*1 out of 5 wMP samples showed a peak at 165 °C

Table S2: Crystallinity of wMP and segregated recycled thermoplastics calculated from DSC analysis.

Sample	Crystallinity (%)	Average Crystallinity (%)
wMP 1	40.6	40.4
wMP 2	40.3	
wMP 3	40.3	
rLDPE 1	38.9	39.1
rLDPE 2	39.2	
rLLDPE 1	38.6	36.7
rLLDPE 2	34.8	
rHDPE 1	75.1	76.8
rHDPE 2	78.5	
rPP 1	51.2	54.1
rPP 2	57.0	

vLDPE 1	37.9	38.6
vLDPE 2	39.2	

Table S3: Onset and endset temperatures for thermal degradation of wMP and recycled plastics.

Sample	Degradation Onset (ºC)	Degradation Endset (ºC)
wMP	350	485
rLDPE	390	487
rLLDPE	380	495
rHDPE	410	490
rPP	380	475
vLDPE	400	485

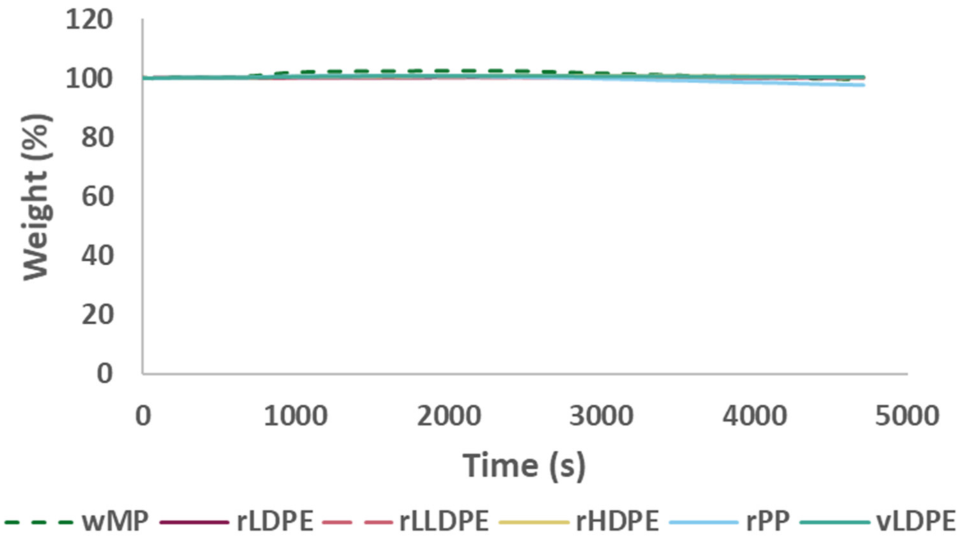

Figure S2: Change in weight of the samples after holding at 200 ºC for 1 hour in air.

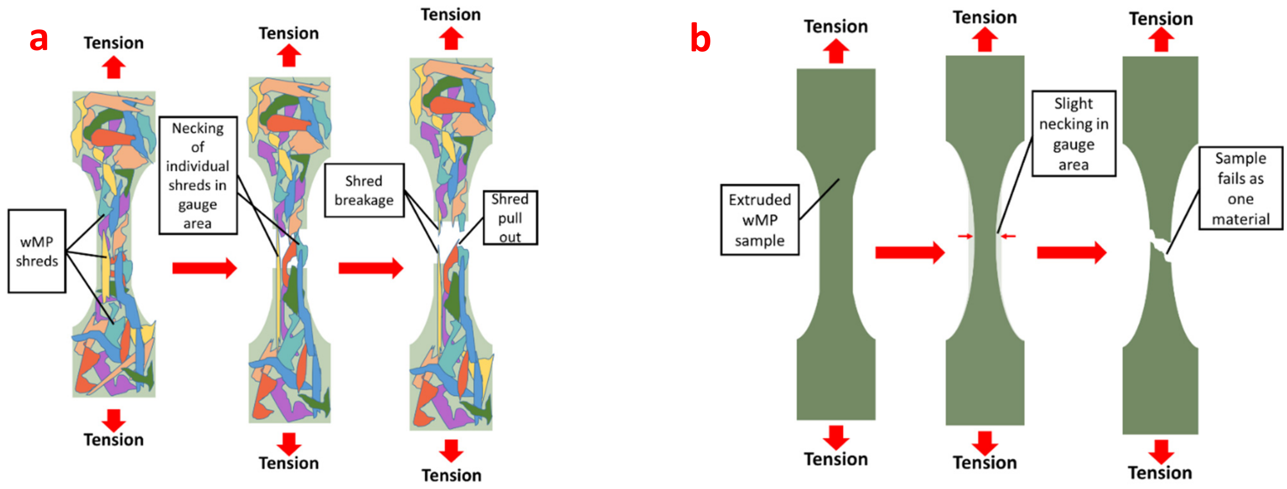

Figure S3: Schematic showing the failure behaviour in (a) wMP-2 and (b) wMP-2-ex during tensile testing.

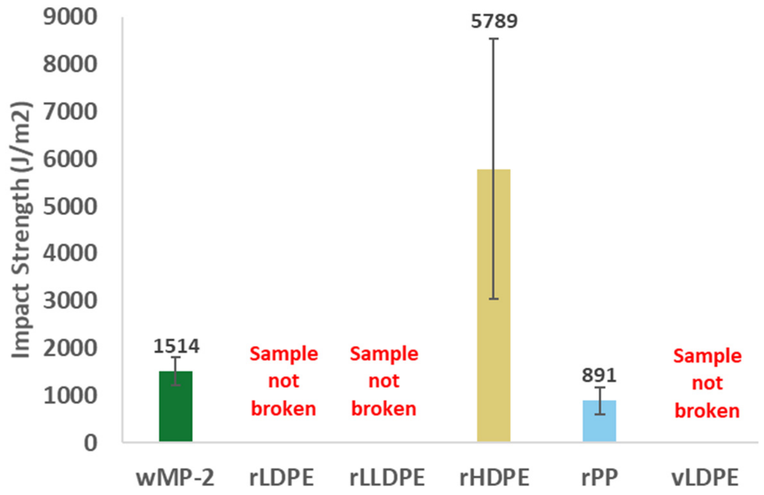

Figure S4: Izod impact strength (un-notched) of wMP-2 in comparison to recycled plastics (rPP and rHDPE).

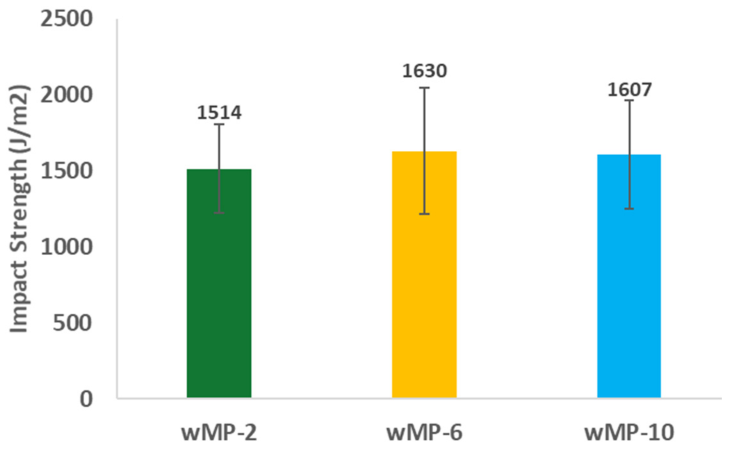

Figure S5: Comparison of Izod impact strength (un-notched) of wMP manufactured under different pressures.

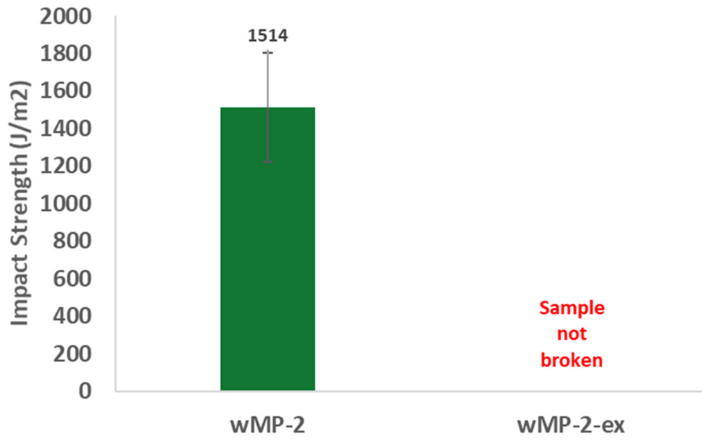

Figure S6: Comparison of Izod impact strength (un-notched) of wMP samples produced by compression moulding of wMP shreds and by compression moulding of an extruded block of wMP.

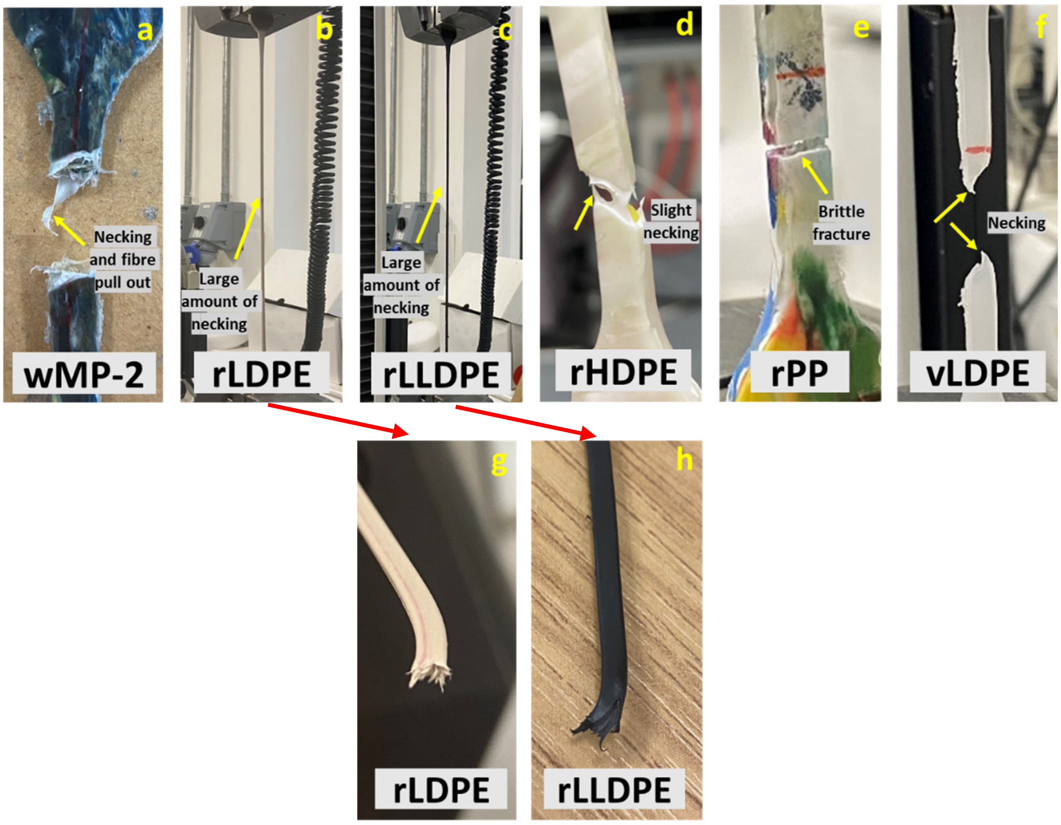

Figure S7: Representative tensile test specimens of (a) wMP-2, (b) rLDPE, (c) rLLDPE, (d) rHDPE, (e) rPP and (f) vLDPE during tensile testing with additional close up images of (g) rLDPE and (h) rLLDPE, due to the high amount of stretching.

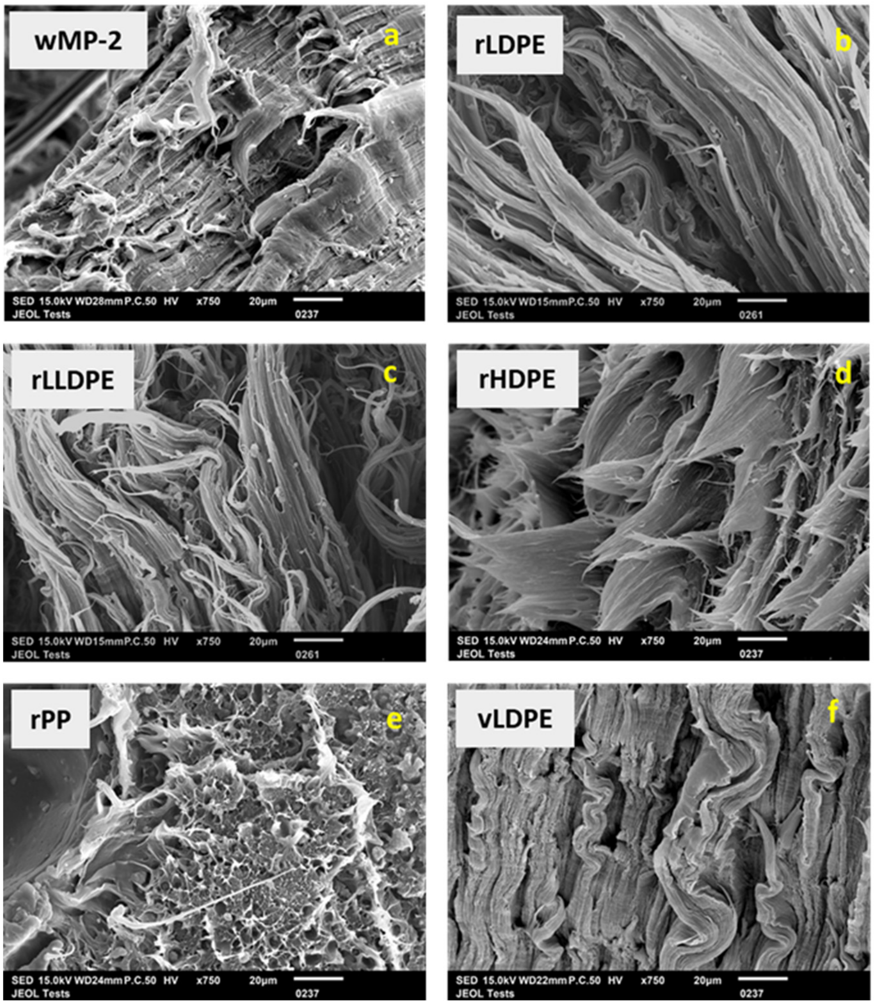

Figure S8: SEM images of the fracture surface of tested tensile specimens at x750 magnification.

1. Selke, S.E. and R.J. Hernandez, *Packaging: Polymers for Containers*, in *Encyclopedia of Materials: Science and Technology*, K.H.J. Buschow, et al., Editors. 2001, Elsevier: Oxford. p. 6646-6652.
2. Jin, H., et al., *The effect of extensive mechanical recycling on the properties of low density polyethylene.* Polymer Degradation and Stability, 2012. **97**(11): p. 2262–2272.
3. Jordan, J., et al., *Mechanical Properties of Low Density Polyethylene.* Journal of Dynamic Behavior of Materials, 2016. **2**.

4. Mohammadi, H., M. Vincent, and H. Marand, *Investigating the equilibrium melting temperature of linear polyethylene using the non-linear Hoffman-Weeks approach.* Polymer, 2018. **146**: p. 344-360.

5. Menyhárd, A., J.D. Menczel, and T. Abraham, *12 - Polypropylene fibers*, in *Thermal Analysis of Textiles and Fibers*, M. Jaffe and J.D. Menczel, Editors. 2020, Woodhead Publishing. p. 205-222.
